# The Risk of Bladder Cancer in Type 2 Diabetes Mellitus with Combination Therapy of SGLT-2 Inhibitors and Pioglitazone

**DOI:** 10.3390/jpm11090828

**Published:** 2021-08-24

**Authors:** Yan-Rong Li, Chi-Hung Liu, Wei-Chiao Sun, Pei-Yi Fan, Feng-Hsuan Liu, Tien-Hsing Chen, Victor Chien-Chia Wu, Chihung Lin, Ching-Chung Hsiao

**Affiliations:** 1 Division of Endocrinology and Metabolism, Department of Internal Medicine, Linkou Chang Gung Memorial Hospital, Taoyuan 333, Taiwan; mr8252@cgmh.org.tw (Y.-R.L.); a122liu@cgmh.org.tw (F.-H.L.); 2 College of Medicine, Chang Gung University, Taoyuan 333, Taiwan; ivanliu0519@gmail.com; 3 Stroke Center and Department of Neurology, Linkou Chang Gung Memorial Hospital, Taoyuan 333, Taiwan; 4 Department of Nephrology, New Taipei Municipal TuCheng Hospital, New Taipei City 236, Taiwan; harmonica2nd@gmail.com (W.-C.S.); glorydmoment@gmail.com (P.-Y.F.); 5 Division of Cardiology, Keelung Chang Gung Memorial Hospital, Keelung 204, Taiwan; skyheart0826@gmail.com; 6 Division of Cardiology, Linkou Chang Gung Memorial Hospital, Taoyuan 333, Taiwan; victorcwu@hotmail.com; 7 Center for Artificial Intelligence in Medicine, Linkou Chang Gung Memorial Hospital, Taoyuan 333, Taiwan

**Keywords:** type 2 diabetes mellitus, bladder cancer, sodium glucose cotransporter-2 (SGLT-2) inhibitors, pioglitazone, mortality

## Abstract

**Background:** Either sodium-glucose cotransporter-2 (SGLT-2) inhibitors or pioglitazone (Pio) has doubtful issues of bladder cancer, especially for the combination therapy with these two drugs. Our study aimed to investigate the risk of bladder cancer under combination therapy of SGLT-2 inhibitors and Pio. **Materials and Methods:** We included 97,024 patients with type 2 diabetes mellitus (T2DM) in the Chang Gung Research Database in Taiwan from 1 January 2016 to 31 December 2019. The primary outcome was newly diagnosed bladder cancer after combination therapy with SGLT-2 inhibitors and Pio. Group 1 received both study drugs, group 2 received SGLT-2 inhibitors, group 3 received Pio, and group 4 received non-study drugs (the reference group). The secondary outcome in each group was all-cause mortality. Results: In group 1, no newly diagnosed bladder cancer was detected after a mean 2.8-year follow-up and all-cause mortality decreased significantly (adjusted hazard ratio (AHR), 0.70; 95% confidence interval (CI), 0.54–0.92) in comparison to the reference group (group 4). In group 2 and group 3, no trend of increased bladder cancer was observed (group 2: AHR 0.49, 95% CI 0.05–4.94; group 3: AHR 0.48, 95% CI 0.15–1.58) and it still reduced all-cause mortality (group 2: AHR 0.83, 95% CI 0.70–0.99; group 3: AHR 0.90, 95% CI 0.83–0.99). **Conclusions:** In T2DM patients without previous or active bladder cancer, the combination therapy of SGLT-2 inhibitors and Pio was not associated with newly diagnosed bladder cancer and had lower all-cause mortality.

## 1. Introduction

Type 2 diabetes mellitus (T2DM) is a major risk factor of cardiac diseases, and it is considered a “Coronary Heart Disease Equivalent” [1]. On the other hand, heart failure (HF) is highly prevalent in patients with T2DM. The mortality rate among patients with T2DM who develop HF has been found to be 32.7% per year (hazard ratio: 10.6) [2]. In addition, patients with diabetes have around twice the risk of ischemic cerebral infarction compared to those without diabetes [3]. As a result, when treating T2DM, in addition to glycemic control, anti-hyperglycemic agents that can lower the risks of cardiovascular (CV) diseases and HF should be prioritized.

Sodium-glucose cotransporter-2 (SGLT-2) inhibitors reduce glucose reabsorption through the renal system in a mechanism independent of insulin. Randomized clinical trials (RCTs) of SGLT-2 inhibitors, such as empagliflozin, canagliflozin, and dapagliflozin, have shown that these drugs not only lower plasma glucose concentration, but also have CV benefits [4,5,6] and reduce the risk of HF [7,8]. Pioglitazone (Pio), a molecule of the thiazolidinedione (TZD) class, is a potent peroxisome proliferator-activated receptor-γ (PPAR-γ) agonist with protective vascular effects [9,10]. Pio could reduce the risks of recurrent stroke in patients with insulin resistance or T2DM [9,10], but it leads to an increased risk of HF [11]. Therefore, combination therapy with SGLT-2 inhibitors and Pio is reasonable because these two kinds of anti-hyperglycemic agents have CV benefits, whereas SGLT-2 inhibitors may lower the risks of HF associated with Pio. However, either SGLT-2 inhibitors or Pio have doubtful issues of increasing the risks of bladder cancer [12,13]. Whether the combination therapy with SGLT-2 inhibitors and Pio will increase the risks of bladder cancer remains unknown. Therefore, this real-world cohort study investigated whether combination therapy with SGLT-2 inhibitors and Pio in T2DM patients is associated with an increased risk of bladder cancer.

## 2. Materials and Methods

### 2.1. Data Source

A retrospectively cohort study was carried out by analyzing data from the Chang Gung Research Database (CGRD) from 1 January 2016 to 31 December 2019. The study protocol was approved by the Institutional Review Board, Chang Gung Medical Foundation, Taiwan (IRB No.: 202100656B0). CGRD, the largest multi-institutional electronic medical records database in Taiwan, is a databank from the Chang Gung Memorial Hospital medical system containing data from two medical centers, two regional hospitals, and three district hospitals with a total of 10,050 beds and approximately 280,000 admissions per year, including all visits to the outpatient departments, emergency departments, and hospitalizations of seven main branch hospitals with a nationwide distribution (covering about 1.3 million individuals, 6% of the population in Taiwan) [14]. The identifications and registrations of diseases in the CGRD were based on the International Classification of Diseases, Ninth Revision, Clinical Modification (ICD-9-CM) before 2016 and the International Classification of Diseases, Tenth Revision, Clinical Modification (ICD-10-CM) afterward. There have been some studies from the CGRD that have provided evidence of valid assessment and treatment outcomes [14,15,16,17]. Because each enrolled patient had an encryption procedure, the informed consent was waived for the present study.

In Taiwan’s national insurance program, patients with specific chronic conditions, including type 1 diabetes mellitus (T1DM), dialysis and kidney transplant patients, and malignancies, are qualified for a catastrophic illness certificate (Registry for Catastrophic Illness Patient Database (RCIPD)). To qualify for a certificate, a patient’s condition must be repeatedly verified by a peer review group based on clinical evidence, pathological findings, and laboratory data. CGRD had been connected to RCIPD and included the information about catastrophic illnesses.

### 2.2. Patient Selection and Study Design

**Figure 1:**
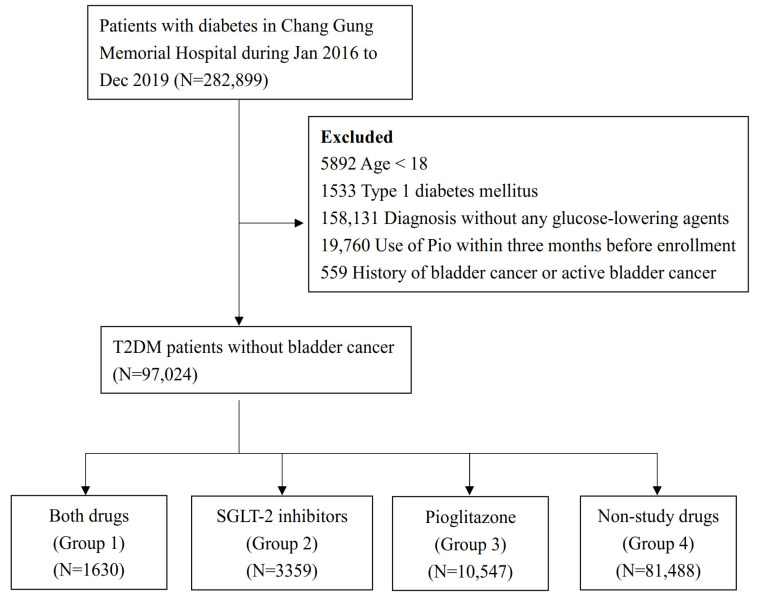
Flowchart of patient selection.

Figure 1 shows the flowchart of the study design and patient enrollment. We retrospectively searched the CGRD for patients with a diagnosis of diabetes from 1 January 2016 to 31 December 2019 (N = 282,899). Using information about catastrophic illnesses, we excluded patients with T1DM (N = 1533). Patients aged less than 18 years were also excluded (N = 5892). To evaluate the association between the study drugs and newly diagnosed bladder cancer, T2DM patients with previous or active bladder cancer were also excluded from the present study (N = 559). The diagnosis of T2DM was defined by at least two outpatient claims or one inpatient claim with ICD-9-CM code = 250 or ICD-10-CM code = E11 and the use of at least one of the oral anti-diabetic agents, including metformin, sulfonylurea, glinides, TZD, acarbose, dipeptidylpeptidase-4 inhibitors, glucagon-like peptide-1receptor agonist, SGLT-2 inhibitors, or insulin. We excluded patients who were not using glucose-lowering agents (N = 158,131) as not having been validly diagnosed with T2DM. Besides, because TZD was one of our main study drugs, patients who had used Pio within three months before enrollment into the study (N = 19,760) were also excluded. Finally, 97,024 T2DM patients were enrolled in our study.

### 2.3. Exposure to the Study Drug

Since SGLT-2 inhibitors were first introduced in Taiwan in 2015 and were available in our hospital from 2016, we defined the index date of group 1–3 as the date of first prescription of SGLT-2 inhibitors and/or Pio and the index date of group 4 as the date of first prescription of other anti-hyperglycemic agents between 1 January 2016 and 31 December 2019. The definition of exposure to study drugs in the present study was adopted from the previously published pharmaco-epidemiological studies for the evaluation of drug effectiveness and adverse events [18,19,20,21]. Because SGLT-2 inhibitors and Pio were our main study drugs, the eligible T2DM patients were divided into four groups based on their use of these two drugs. The first group (group 1) consisted of patients with T2DM on both study drugs, the second group (group 2) those with T2DM on SGLT-2 inhibitors, the third group (group 3) those with T2DM on Pio, and the fourth group (group 4, the reference group) those with T2DM on non-study drugs. The follow-up period was defined as the period from the index date until the date of the diagnosis of bladder cancer, discontinuation of study drugs, loss to follow-up, death, or the end of the study period (31 December 2019), whichever occurred first.

### 2.4. Covariates

The patients’ characteristics, such as age, gender, duration of having diabetes, hospital levels of admission, major baseline co-morbidities, and main medications (CV, renal and anti-hyperglycemic agents), were collected. The co-morbidity was defined as two outpatient diagnoses or one discharge diagnosis within one year before enrollment. Most diagnostic codes of these co-morbidities have been validated in previous national database studies [15]. The baseline medications were identified according to the claims data within three months before the index date. The major baseline laboratory data, such as hemoglobin A1c, renal function tests (serum creatinine and estimated glomerular filtration rate), and liver enzymes (ALT), were also analyzed.

### 2.5. Outcome Measurement

The primary outcome was the cumulative incidence of newly diagnosed bladder cancer in group 1, group 2, group 3, and group 4 (the reference group) during a mean follow-up period of 2.8 years. The secondary outcome in each group was all-cause mortality. Death was considered a competing risk of the onset of bladder cancer.

### 2.6. Statistical Analysis

To balance the baseline characteristics and compare the risks of the outcomes among multiple groups, inverse probability of treatment weighting (IPTW) for propensity scores (PSs) was performed. Table 1 shows the variables before IPTW, and Table 2 shows the variables after IPTW. The follow-up year in Table 1 was replaced with the index date for the IPTW. An inverse probability of treatment weight is a synthetic population in which treatment assignment is independent of the baseline covariates. It generates more homogeneous groups for comparison. The balance among the multiple treatment groups before and after IPTW was assessed using the absolute standardized mean difference (ASMD), in which an absolute value less than 0.2 indicates a small difference between groups.

Kaplan–Meier curve analysis was used to estimate the cumulative incidence of newly diagnosed bladder cancer for each group and the difference among the groups was assessed using the log-rank test. The covariates were also subjected to multivariate analyses with a Cox proportional-hazards model and adjusted using the IPTW method, which was based on the selected covariates listed in Table 1. Table 2 shows four variables with a larger than 0.2 standardized mean difference after weighting (chronic kidney disease, sulfonylurea, HbA1c, and creatinine). The competing risk analysis was based on considering death as a competing risk of the incidence of newly diagnosed bladder cancer. A two-sided *p* value < 0.05 defined statistical significance. Analyses were performed using SAS software (version 9.4; SAS Institute Inc., Cary, NC, USA).

## 3. Results

### 3.1. Study Patients

Between 1 January 2016 and 31 December 2019, we screened 282,899 diabetic patients. After exclusion of patients with T1DM, those aged less than 18 years old, T2DM patients without anti-hyperglycemia agents, those who had used Pio within three months before enrollment, and those with previous or active bladder cancer, a total of 97,024 subjects were enrolled and eligible for the final analyses. The eligible T2DM patients were divided into four groups based on their use of two main study drugs: SGLT-2 inhibitors and Pio, respectively. Group 1 (N = 1630) was T2DM with both study drugs, group 2 (N = 3359) was T2DM with SGLT-2 inhibitors, group 3 (N = 10,547) was T2DM with Pio, and group 4, the reference group (N = 81,488), was T2DM with non-study drugs (Figure 1).

### 3.2. Baseline Characteristics

Table 1 shows the wide range of baseline comorbidities of the study subjects, their important medications, and the major laboratory data. Table 2 shows the variables after IPTW to minimize potential selection bias and to make our study groups well-balanced when comparing treatment effects. After IPTW, most baseline characteristics and medications were well balanced among these four groups except for chronic kidney disease (higher in group 3, 11.5%), sulfonylurea (higher in group 1, 64.3%), HbA1c (higher in group 1, 8.1%), and serum creatinine (higher in group 3, 1.4mg/dl). The mean age of the entire cohort was 56.8 years (standard deviation (SD) = 11years). The most common co-morbidity was hypertension (60.7–63.7%), followed by dyslipidemia (48.6–53.5%), previous stroke (12.4–15.7%), and coronary artery disease (13.5–14.2%) in each group.

### 3.3. Primary and Secondary Outcomes

**Figure 2:**
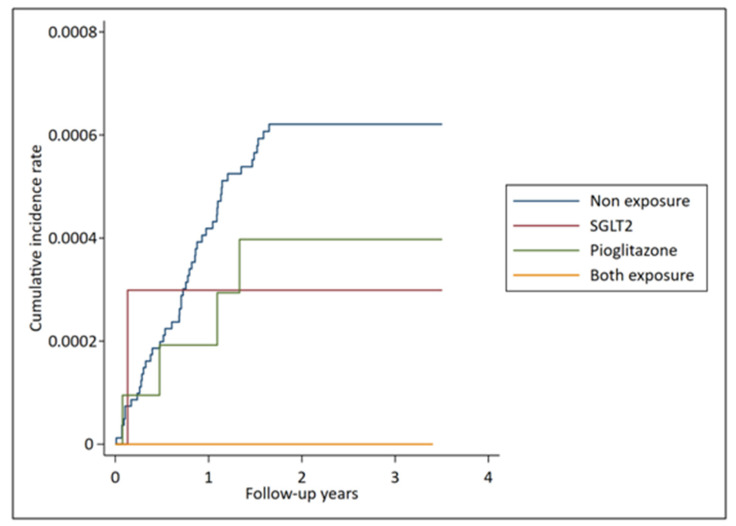
Cumulative incidence of newly diagnosed bladder cancer in each group.

The primary outcome was cumulative incidence of newly diagnosed bladder cancer, and the secondary outcome was all-cause mortality in each group (Figure 2 and Table 3). In group 1, no newly diagnosed bladder cancer was detected after a mean follow-up period of 2.8 years and there was significantly decreased all-cause mortality (adjusted hazard ratio (AHR), 0.70; 95% confidence interval (CI), 0.54–0.92) on comparison with the reference group (group 4). In group 2 and group 3, no trend of increased bladder cancer was observed (group 2: AHR 0.49, 95% CI 0.05–4.94; group 3: AHR 0.48, 95% CI 0.15–1.58) and it still reduced all-cause mortality (group 2: AHR 0.83, 95% CI 0.70–0.99; group 3: AHR 0.90, 95% CI 0.83–0.99).

## 4. Discussion

In this observational cohort study from the largest multi-institutional databank in Taiwan, we investigated whether combination therapy of SGLT-2 inhibitors and Pio in T2DM patients is associated with an increased risk of bladder cancer. After including 97,024 T2DM patients without previous or active bladder cancer and with follow-up for 2.8 years, the combination therapy of SGLT-2 inhibitors and Pio was not found to be associated with newly diagnosed bladder cancer in this short-term research from CGRD. Besides, the individual use of SGLT-2 inhibitors and Pio was also not found to be associated with newly diagnosed bladder cancer when compared to other anti-hyperglycemic agents. The Food and Drug Administration in the United States (U.S. FDA) has previously commented on the need for post-marketing surveillance studies for issues of bladder cancer related to SGLT-2 inhibitors based on the concern about chronic exposure of urinary tract with glycosuria [12]. Therefore, the strength of the present study is that it is the first real-world large cohort study in Asia to investigate the risk of bladder cancer in patients on combination therapy of SGLT-2 inhibitors and Pio and the results showed neutral effects with short-term use. Of note, this combination regimen was associated with reduced all-cause mortality.

In terms of T2DM with SGLT-2 inhibitors, our results revealing neutral effects on newly diagnosed bladder cancer are compatible with those of a previous meta-analysis of large randomized controlled trials, which showed that SGLT-2 inhibitors had no significantly increased risks of bladder cancer [22]. However, a recent case-control study in a European database showed a disproportionately high number of cases of bladder cancer occurred in patients with SGLT-2 inhibitors [23]. There are several explanations for these discrepant findings. First, for evidence-based medicine, meta-analysis of relevant RCTs and the result from a large cohort study are more convincing than a case-control study. Moreover, the European study was a pharmacovigilance study, which does not quantify the risk or prove the causality, and it was affected by undernotification. Second, our results are of a study in an Asian population, which could make a difference. Third, unlike the aforementioned European study, which enrolled patients with a history of bladder cancer, in our cohort study, patients with previous or active bladder cancer were excluded. As a result, the events of recurrent bladder cancer would not be observed and then the total number of bladder cancer in the final outcome would be less. Fourth, our study had a relatively short period for the observation of the effects on carcinogenesis.

In terms of T2DM with Pio, the issues of bladder cancer are still uncertain due to the conflicting and controversial results from previous studies [24,25,26]. In the present study, the risk of newly diagnosed bladder cancer in the Pio groups did not increase. However, the U.S. FDA has previously mentioned that Pio should not be administered to patients with active bladder cancer [10]. In our study, patients with previous or active bladder cancer were not enrolled. Therefore, the interpretation of our results was that for T2DM patients without previous or active bladder cancer, short-term use of Pio is not associated with an increased risk of bladder cancer.

In terms of reducing all-cause mortality, our results were compatible with the findings from a recent meta-analysis of RCTs, which revealed that SGLT-2 inhibitors had a significantly lower rate of all-cause mortality (OR 0.85, 95% CI, 0.80–0.91, *p* < 0.00001). With a random-effects model, especially with a larger Asian population included, there was a higher reduction in all-cause mortality [27]. Besides, for T2DM patients with a high CV disease risk, the PROspectivepioglitAzone Clinical Trial In macroVascular Events (PROactive) study showed that Pio could reduce 16% of the secondary composite outcome, including all-cause mortality, non-fatal myocardial infarction, and stroke [28]. As a result, it can be expected that the combination therapy of SGLT-2 inhibitors and Pio could lower all-cause mortality further than those without these two drugs.

This present study had some limitations. First, although we tried our best to include a wide range of baseline comorbidities and medications with the IPTW method to minimize potential selection bias and to make our study groups well-balanced when comparing treatment effects, for a retrospective cohort study, there is always a possibility of selection bias. Second, drug adherence could have had an influence on the study results, and in a real-world study, we can only presume drug adherence based on prescription records. Third, the generalizability of our conclusions to other ethnicities and all kinds of SGLT-2 inhibitors remains uncertain because the present study was conducted in an Asian population and only three kinds of SGLT-2 inhibitors (empagliflozin, canagliflozin, and dapagliflozin) were included in our study. Lastly, the present retrospective observational cohort study was unable to define a causal relationship due to potential selection bias and unmeasured confounding factors; therefore, future RCTs will be needed to confirm our findings, especially for the long-term effects. However, RCTs are not always feasible due to the fact that they are time-consuming and involve high costs as well as issues of ethical considerations. Therefore, our real-world post-marketing cohort study is still valuable to answer unknown questions.

## 5. Conclusions

In summary, for T2DM patients without previous or active bladder cancer, the combination therapy of SGLT-2 inhibitors and Pio was not associated with an increased risk of newly diagnosed bladder cancer and had a lower risk of all-cause mortality during a mean observation period of 2.8 years. The combination therapy of SGLT-2 inhibitors and Pio is reasonable because these two anti-hyperglycemic agents have CV benefits and could decrease all-cause mortality.

## Figures and Tables

**Table 1:** Demographic characteristics of the study population before propensity score weighting.

	Before Weighting				ASMD
Variable	Both Drugs (N = 1630)	SGLT-2 Inhibitors (N = 3359)	Pioglitazone (N = 10,547)	Non-Study Drugs (N = 81,488)	
Age, years	49.3 ± 10.5	50.3 ± 10.8	54.5 ± 10.5	58.0 ± 11.7	0.78
Male sex	959 (58.8)	1839 (54.8)	5958 (56.5)	42,629 (52.3)	0.13
Hospital level					0.17
Medical center	1044 (64.1)	1677 (49.9)	6725 (63.8)	45,367 (55.7)	
Regional/district hospital	586 (36.0)	1682 (50.1)	3822 (36.2)	36,121 (44.3)	
Diabetes duration, year	10.5 ± 4.5	9.5 ± 4.6	10.8 ± 4.5	8.9 ± 4.7	0.41
CCI					0.41
0	1044 (64.1)	1923 (57.3)	5681 (53.9)	36,368 (44.6)	
1	312 (19.1)	823 (24.5)	2273 (21.6)	19,425 (23.8)	
2+	274 (16.8)	613 (18.3)	2593 (24.6)	25,695 (31.5)	
Comorbidity					
Previous stroke	98 (6.0)	205 (6.1)	1286 (12.2)	11,595 (14.2)	0.28
Gout	64 (3.9)	152 (4.5)	512 (4.9)	5061 (6.2)	0.10
Hypertension	953 (58.5)	2009 (59.8)	6522 (61.8)	50,879 (62.4)	0.08
Previous myocardial infarction	26 (1.6)	139 (4.1)	219 (2.1)	2767 (3.4)	0.12
Coronary artery disease	249 (15.3)	535 (15.9)	1303 (12.4)	11,573 (14.2)	0.05
Chronic kidney disease	39 (2.4)	87 (2.6)	1018 (9.7)	8292 (10.2)	0.32
COPD	43 (2.6)	93 (2.8)	337 (3.2)	3761 (4.6)	0.11
Dyslipidemia	974 (59.8)	2017 (60.1)	5928 (56.2)	39,531 (48.5)	0.23
Hx of malignancy (except for bladder cancer)	91 (5.6)	217 (6.5)	730 (6.9)	8298 (10.2)	0.17
Anti-hypertensive agents					
ACEI/ARB	1030 (63.2)	2147 (63.9)	6892 (65.4)	48,240 (59.2)	0.13
CCB	346 (21.2)	767 (22.8)	2878 (27.3)	26,386 (32.4)	0.25
Alpha-blocker	123 (7.6)	291 (8.7)	1249 (11.8)	11,224 (13.8)	0.20
Beta-blocker	513 (31.5)	1105 (32.9)	3216 (30.5)	26,045 (32.0)	0.03
Thiazide	48 (2.9)	68 (2.0)	310 (2.9)	2058 (2.5)	0.03
Loop diuretics	88 (5.4)	232 (6.9)	1097 (10.4)	9848 (12.1)	0.24
Spironolactone	30 (1.8)	102 (3.0)	233 (2.2)	2417 (3.0)	0.07
Anti-diabetic agents					
Biguanide (Metformin)	1257 (77.1)	2009 (59.8)	5950 (56.4)	38,961 (47.8)	0.63
Sulfonylurea	1054 (64.7)	2175 (64.8)	6845 (64.9)	37,759 (46.3)	0.38
Glinide	60 (3.7)	114 (3.4)	553 (5.2)	4952 (6.1)	0.13
DPP-4 I	1222 (75.0)	2348 (69.9)	6066 (57.5)	35,657 (43.8)	0.67
GLP-1 RA	28 (1.7)	74 (2.2)	129 (1.2)	445 (0.6)	0.14
Insulin	303 (18.6)	1088 (32.4)	1964 (18.6)	19,915 (24.4)	0.18
Alpha glucosidase inhibitors	467 (28.7)	823 (24.5)	2243 (21.3)	11,323 (13.9)	0.37
Other medications					
Aspirin	576 (35.3)	995 (29.6)	3571 (33.9)	24,275 (29.8)	0.12
Clopidogrel	66 (4.1)	220 (6.6)	709 (6.7)	6915 (8.5)	0.18
Cilostazol	25 (1.5)	59 (1.8)	262 (2.5)	2495 (3.1)	0.10
Statin	987 (60.6)	1998 (59.5)	6331 (60.0)	40,648 (49.9)	0.22
Fibrate	177 (10.9)	373 (11.1)	1052 (10.0)	6909 (8.5)	0.09
NSAIDs	252 (15.5)	665 (19.8)	2055 (19.5)	19,607 (24.1)	0.22
Steroid	169 (10.4)	396 (11.8)	1524 (14.5)	15,320 (18.8)	0.24
Lab (baseline)					
HbA1c, %	8.5 ± 1.5	8.5 ± 1.6	7.7 ± 1.5	7.4 ± 1.4	0.73
Creatinine	0.9 ± 0.3	0.9 ± 0.3	1.3 ± 1.3	1.3 ± 1.6	0.39
eGFR	78.7 ± 17.3	78.2 ± 18.0	65.9 ± 23.8	65.4 ± 24.5	0.63
ALT	29.3 ± 22.7	32.9 ± 30.7	26.0 ± 37.0	29.0 ± 31.9	0.13
Follow-up year	2.8 ± 0.8	3.0 ± 0.6	2.9 ± 0.8	2.9 ± 0.7	0.21

ACEI, angiotensin converting enzyme inhibitors; ALT, alanine transaminase; ARB, angiotensin receptor blockers; ASMD, absolute value of standardized mean difference; CCB, calcium channel blockers; COPD, chronic obstructive pulmonary disease; DPP-4 I, dipeptidyl peptidase-4 inhibitors; NSAID, non-steroidal anti-inflammatory drugs; eGFR estimated glomerular filtration rate; GLP-1 RA, glucagon-like peptide-1 receptor agonist; HbA1c, hemoglobin A1c; SGLT-2, sodium glucose co-transporters 2. Data presented as frequency (percentage) or mean ± standard deviation.

**Table 2:** Demographic characteristics of the study population after propensity score weighting.

	After Weighting				ASMD
Variable	Both Drugs (N =1630)	SGLT-2 Inhibitors (N =3359)	Pioglitazone (N =10,547)	Non-Study Drugs (N =81,488)	
Age, years	56.5 ± 10.5	56.1 ± 10.7	57.2 ± 11.3	57.2 ± 11.8	0.10
Male sex	55.2%	54.0%	54.4%	53.0%	0.04
Hospital level					0.12
Medical center	50.4%	58.5%	53.5%	56.5%	
Regional/district hospital	49.6%	41.5%	46.5%	43.5%	
Diabetes duration, year	8.9 ± 4.5	8.9 ± 4.6	9.1 ± 4.8	9.2 ± 4.7	0.05
CCI					0.13
0	50.4%	48.2%	44.7%	46.5%	
1	25.1%	23.7%	24.3%	23.5%	
2+	24.5%	28.2%	31.0%	30.0%	
Comorbidity					
Previous stroke	15.7%	12.4%	13.4%	13.6%	0.06
Gout	7.8%	9.3%	5.6%	6.0%	0.13
Hypertension	60.7%	60.2%	63.7%	62.2%	0.04
Previous myocardial infarction	2.9%	3.8%	3.3%	3.3%	0.03
Coronary artery disease	13.8%	13.5%	14.2%	14.1%	0.02
Chronic kidney disease	3.4%	8.2%	11.5%	9.7%	0.26
COPD	6.3%	3.5%	4.9%	4.4%	0.09
Dyslipidemia	53.5%	48.6%	49.0%	49.9%	0.07
Hx of malignancy (except for bladder cancer)	6.1%	7.5%	9.1%	9.6%	0.13
Anti-hypertensive agents					
ACEI/ARB	60.7%	56.7%	62.0%	60.1%	0.07
CCB	29.5%	32.3%	32.5%	31.3%	0.04
Alpha-blocker	14.2%	13.2%	14.3%	13.3%	0.03
Beta-blocker	33.1%	29.5%	33.3%	31.9%	0.05
Thiazide	2.8%	2.7%	2.3%	2.6%	0.02
Loop diuretics	11.6%	12.2%	12.4%	11.6%	0.02
Spironolactone	4.2%	3.3%	3.1%	2.9%	0.07
Anti-diabetic agents					
Biguanide (Metformin)	41.4%	43.1%	46.6%	49.7%	0.17
Sulfonylurea	64.3%	49.6%	51.5%	49.5%	0.30
Glinide	2.8%	6.2%	7.0%	5.9%	0.15
DPP-4 I	42.6%	43.7%	47.8%	46.9%	0.09
GLP-1 RA	0.9%	0.8%	0.8%	0.7%	0.02
Insulin	26.9%	27.7%	26.3%	24.2%	0.08
Alpha glucosidase inhibitors	15.4%	19.5%	16.9%	15.4%	0.11
Other medications					
Aspirin	27.5%	29.4%	30.6%	30.3%	0.06
Clopidogrel	11.8%	6.4%	8.6%	8.2%	0.12
Cilostazol	2.6%	3.1%	3.4%	2.9%	0.03
Statin	52.5%	48.3%	52.7%	51.5%	0.07
Fibrate	12.5%	8.5%	9.1%	8.8%	0.12
NSAIDs including	25.6%	22.4%	24.5%	23.3%	0.05
Steroid	16.0%	17.7%	19.8%	17.9%	0.05
Lab (baseline)					
HbA1c, %	8.1 ± 1.3	8.0 ± 1.8	7.6 ± 1.7	7.5 ± 1.6	0.42
Creatinine	1.0 ± 0.4	1.2 ± 1.0	1.4 ± 1.9	1.3 ± 1.6	0.24
eGFR	67.8 ± 19.4	66.2 ± 23.4	65.4 ± 25.8	66.2 ± 24.4	0.07
ALT	30.6 ± 22.1	30.0 ± 23.7	52.9 ± 223.5	28.9 ± 30.6	0.15
Follow-up year	2.8 ± 0.8	2.8 ± 0.8	2.8 ± 0.8	2.9 ± 0.7	0.06

ACEI, angiotensin converting enzyme inhibitors; ALT, alanine transaminase; ARB, angiotensin receptor blockers; ASMD, absolute value of standardized mean difference; CCB, calcium channel blockers; COPD, chronic obstructive pulmonary disease; DPP-4 I, dipeptidyl peptidase-4 inhibitors; NSAID, non-steroidal anti-inflammatory drugs; eGFR estimated glomerular filtration rate; GLP-1 RA, glucagon-like peptide-1 receptor agonist; HbA1c, hemoglobin A1c; SGLT-2, sodium glucose co-transporters 2. Data presented as frequency (percentage) or mean ± standard deviation.

**Table 3:** Risks of bladder cancer and all-cause mortality according to exposure of study drugs.

Exposure Status	No. of Patients	No. of Patients with Bladder Cancer (%)		AHR (95% CI) ^ab^ for Bladder Cancer	*p* Value	No. of Death (%)		AHR (95% CI) ^a^ for Death	*p* Value
No exposure	81,488	48	(0.06%)	1.00 (Reference)		4846	(5.95%)		
SGLT-2 I	3359	1	(0.03%)	0.49 (0.05–4.94)	0.546	62	(1.85%)	0.83 (0.70–0.99)	0.034
Pioglitazone	10,547	4	(0.04%)	0.48 (0.15–1.58)	0.227	413	(3.92%)	0.90 (0.83–0.99)	0.024
Both drugs	1630	0	(0%)	NA	NA	33	(2.02%)	0.70 (0.54–0.92)	0.010

NA, not applicable; AHR, adjusted hazard ratio; SGLT-2 I, sodium glucose co-transporters 2 inhibitors. ^a^ The adjusted hazard ratios were calculated by the Cox proportional hazard model and adjusted using the inverse probability of treatment weighting method which measured based on the selected covariates listed in Table 1 and four variables with larger than 0.2 of standardized mean difference after weighting (chronic kidney disease, sulfonylurea, HbA1c, and creatinine). ^b^ The competing risk analysis was based on considering the death as a competing risk of the incidence of bladder cancer.

## Data Availability

The datasets generated and/or analyzed during the present study are not completely and publicly available due some of our data are limited to be free accessed due to the IRB regulations of CGRD data in Taiwan. However, these data are available from the corresponding author on reasonable request.
